# Optimization of Ultrasound-Assisted Extraction and Characterization of the Phenolic Compounds in Rose Distillation Side Streams Using Spectrophotometric Assays and High-Throughput Analytical Techniques

**DOI:** 10.3390/molecules28217403

**Published:** 2023-11-02

**Authors:** Thalia Tsiaka, Natalia A. Stavropoulou, Maria C. Giannakourou, Irini F. Strati, Vassilia J. Sinanoglou

**Affiliations:** 1 Laboratory of Chemistry, Analysis & Design of Food Processes, Department of Food Science and Technology, University of West Attica, Agiou Spyridonos, 12243 Egaleo, Greece; tsiakath@uniwa.gr (T.T.); nstavropoulou@uniwa.gr (N.A.S.); estrati@uniwa.gr (I.F.S.); 2 Laboratory of Food Chemistry and Technology, School of Chemical Engineering, National Technical University of Athens, Zografou Campus, 9 Iroon Polytechniou St., 15772 Athens, Greece

**Keywords:** rose byproducts, ultrasound-assisted extraction, Box–Behnken design, total phenolic content, antioxidant/antiradical activity, phenolic fingerprint, attenuated total reflection-Fourier transform infrared spectroscopy, liquid chromatography-tandem mass spectrometry

## Abstract

Lately, the essential oils industry has been one of the most expanding markets globally. However, the byproducts generated after the distillation of aromatic plants and their transformation to novel high-added value products consist of a major up-to-date challenge. Thus, the scope of the current study is the optimization of ultrasound-assisted extraction (UAE) for the recovery of phenolic compounds from rose (*Rosa damascena*) post-distillation side streams using Box–Behnken design. In particular, the highest total phenolic content (TPC) was achieved at 71% *v*/*v* ethanol–water solution, at 25 min, 40 mL/g dry sample and 53% ultrasound power, while ethanol content and extraction time were the most crucial factors (*p*-value ≤ 0.05) for UAE. Both solid (RSB) and liquid (LSB) rose side streams exhibited significant antiradical and antioxidant activities. The interpretation of attenuated total reflection-Fourier transform infrared (ATR-FTIR) spectra confirmed the presence of compounds with properties such as phenolic compounds, phenolic amide derivatives, and alcohols in the extracts. Moreover, the flavonoids naringenin, quercetin, and kaempferol were the major phenolic compounds, identified in the extracts by liquid chromatography-tandem mass spectrometry analysis (LC-MS/MS), followed by gallic, protocatechuic, p-hydroxybenzoic, and rosmarinic acids. Furthermore, the LC-MS/MS results pinpointed the effect of factors other than the extraction conditions (harvesting parameters, climatic conditions, plant growth stage, etc.) on the phenolic fingerprint of RSB extracts. Therefore, RSB extracts emerge as a promising alternative antioxidant agent in food products.

## 1. Introduction

During the last decade, the production of essential oils (EOs) from aromatic plants and flowers has presented a constant increase in order to cater to the demands of various industries, from cosmetic, personal care, and aroma commerce to food additives and bioinsecticides business. In particular, the EOs market is anticipated to reach an 8% increase in 2030, according to the 2023–2030 forecast [1]. However, the EOs processing industry generates an immense amount of side streams, which mainly consist of a liquid fraction, known as hydrolates or hydrosol or floral water, and a solid fraction, which includes the biomass remaining after the distillation process [2,3].

The Mediterranean flora, which shapes a rich and complex mosaic of aromatic herbs and flowers, has been considered, over time, one of the greatest sources for the production of EOs, and while aromatic herbs, such as basil, oregano, lavender, thyme, rosemary, etc., have been thoroughly investigated for their properties and profile in terms of bioactive compounds, there are still missing studies regarding the bioactive extracts of Mediterranean flowers [4,5]. *Rosa damascena* Mill of the *Rosaceae* family is documented as one of the most popular ornamental flowers with acknowledged pharmacological properties, which is widely used in food and perfume sectors. Although there are several studies related to the composition of EOs (i.e., geraniol, citronellol, nerol, etc.) of the raw plant, there is still scarce information regarding the post-distillation byproducts of rose samples, especially the solid residues [6].

Therefore, in order to showcase the importance of introducing sustainable management strategies for the re-use and re-incorporation of the hydrodistillation byproducts in the market circle, it is worth mentioning that only one part of rose EOs is produced from around 3000 parts of rose flowers, while the world consumption of rose EOs is estimated at 3000–4500 kg annually [7,8]. Besides the pernicious environmental effects and economic burden that follows the disposal of plant byproducts, these substrates constitute an abundant source of health-promoting bioactive compounds, such as phenolic compounds and polyphenols, carotenoids, tannins, catechins, and terpenoids [9]. Among them, phenolic compounds are the predominant group of bioactive molecules in plant tissues owing designated beneficial biological effects (i.e., antioxidant, antitumor, anti-aging, antimicrobial activities, cardiovascular, and metabolic effects, stimulation of microbial gut homeostasis, etc.) [10]. Thus, the implementation and optimization of non-conventional extraction techniques (i.e., ultrasound-assisted extraction (UAE), supercritical fluid extraction (SFE), pressurized liquid extraction (PLE)), which provides rich-in-target compounds extracts of high quality in shorter extraction times using lower to zero amounts of organic solvents is of pivotal significance for the recovery of phenolic compounds from plant byproducts [11].

According to recent data, the phenolic-rich extracts of plant byproducts can be incorporated in active packaging materials or they can be used as a natural antimicrobial agent directly in food products, such as meat products, developing novel high-added value components. Hence, the current study brings into focus the optimization of ultrasound-assisted extraction (UAE) in *Rosa damascena* solid byproducts by applying a 27-run Box–Behnken design in order (a) to recover extracts of high phenolic content, (b) to interrelate the antioxidant and antiradical activities of the extracts with their phenolic profile as delivered by attenuated total reflectance-Fourier transform infrared spectroscopy (ATR-FTIR) and liquid chromatography-tandem mass spectrometry (LC-MS/MS), and (c) to evaluate the effect of the extraction conditions on the total phenolic content and on the type of the extracted phenolic compounds.

## 2. Results

### 2.1. Optimization of Ultrasound-Assisted Extraction (UAE) for the Recovery of Phenolic Compounds from Rose Samples

In order to construct the design matrix and generate the regression equation for the prediction of TPC values in rose samples, 27 experimental runs were performed as proposed by the Box–Behnken design using dried rose petals as substrate. The extraction conditions and the total phenolic content of each experimental run are presented in Table S1. All the experiments were performed in randomized order to minimize the possible effect of other factors not included in the model and to eliminate any systematic errors.

Three of the twenty-seven experiments were conducted with all factors constant at the zero (medium) level (Table S1) to evaluate and confirm the model’s precision by calculating the average and standard deviation of these experiments. In particular, the current model was quite precise since the average TPC value and the respective standard deviation of the three similar experiments was 136.7 (±7.6) mg GAE/g dry sample.

At first, a full quadratic model, which included the quadratic, linear, and linear 2-way interaction terms, was selected to fit the data. Then, based on the analysis of variance (ANOVA) results (Table S2), the highly insignificant terms with *p*-values ≥ 0.20 were removed. At this point, it should be underlined that the linear terms of the four factors under optimization, i.e., A, B, C, D, were not omitted even when they had *p*-values ≥ 0.05 in order to provide a hierarchical final model. Finally, UAE extraction was successfully modeled in order to reliably predict the TPC values of rose samples as proven by the coefficient of determination (R^2^), the adjusted R^2^ (R^2^_adj_), the predicted R^2^ (R^2^_pred_), and the lack-of-fit value, which was non-significant (*p*-value ≥ 0.05, Table S2). In particular, over 93% (Table S2) of the variabilities in the value of the TPC can be explained by the selected investigated factors and therefore the produced Box–Behnken model fitted the data well since R^2^ and R^2^_adj_ were close to 1 and their difference was smaller than 0.2 (Table S2). In addition, the model can predict the TPC values of new rose samples in over 85% of the cases (Table S2).

**Figure 1:**
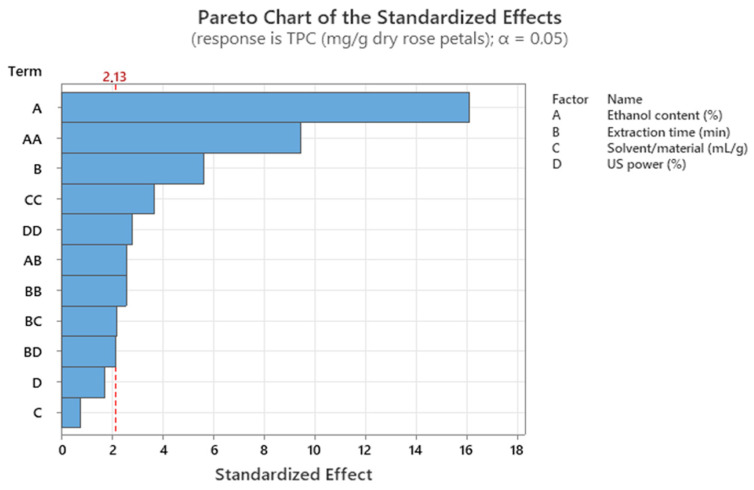
Pareto chart for the Box–Behnken design.

The significance of the model terms in decreasing order is presented in Pareto chart (Figure 1), where all the factors that exceed the vertical red line were considered significant for maximizing the extraction of phenolic compounds from rose samples.

The regression equation that describes the relationship between TPC and ethanol content, extraction time, solvent/material ratio, and US power is presented in Equation (1).

TPC (mg/g dry rose petals) = −718 + 18.28A + 8.12B + 2.62C + 2.545D − 0.1236AA − 0.0597BB − 0.0482CC − 0.01621DD − 0.0520AB + 0.0444BC − 0.0289BD(1)

**Figure 2:**
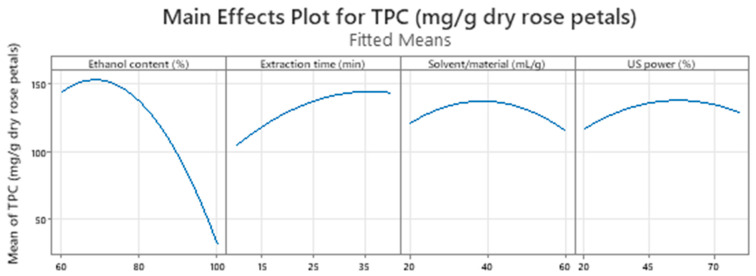
Main effects plots of investigated UAE factors.

**Figure 3:**
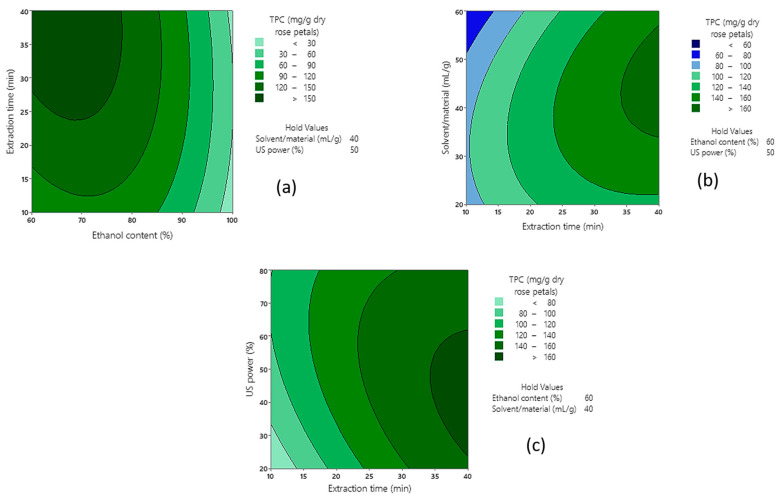
Contour plots for the effect of: (**a**) ethanol content and extraction time; (**b**) extraction time and solvent/material ratio; (**c**) extraction time and US power on TPC of rose extracts.

The main effect (Figure 2) and contour plots (Figure 3) were generated to extract conclusions regarding the values of the examined factors where the TPC of rose extracts was optimized. According to the main effect plots (Figure 2), the steep curvature of the first plots showed that the phenolic content of the rose extracts was affected crucially by the ethanol content, a fact that was also confirmed by the Pareto chart (Figure 1), which appointed this factor as the most important for the recovery of phenolics from rose samples. Specifically, the TPC showed a sheer decrease as the ethanol content was increased, while it presented a relatively mild increase when the extraction lasted longer. On the other hand, the variations of the solvent/material ratio and US power did not exhibit any substantial positive or negative effect on the total content of phenolics, as it was expected based on the *p*-values (*p*-values ≥ 0.05) of the abovementioned factors (Table S2).

Moreover, the 2-way interactions that played a key role in the optimization of the UAE process are presented in the 2D contour plots (Figure 3), where the two factors are plotted pairwise, while the other two are held constant to their medium (0 level) values.

The interpretation of contour plots revealed that the relationship of ethanol content in the solvent solution and extraction time was inverse, since the TPC of the extracts increased at higher extraction times, between 25 and 40 min (greener plot region, Figure 3a) when the extraction solvent contained 60 to 75% ethanol. Higher values of TPC were also observed at the before mentioned extraction times combined with intermediate solvent/material ratios (around 35–45 mL/g) and US power (around 35–55%), as depicted in Figure 3b,c.

Therefore, the optimal UAE conditions proposed by the model were 71% *v*/*v* ethanol content, 25 min, 40 mL/g solvent/material ratio, and 53% US power. The obtained experimental TPC values (141.4 (±5.6) mg GAE/g dry sample) were within the confidence intervals of the predicted theoretical values (152.35 (138.54–166.17) mg GAE/g dry sample) at 95% confidence level. Nonetheless, in the recent literature, the results regarding the TPC of *Rosa damascena* Mill., extracted by classic extraction techniques (i.e., Soxhlet method, heated solid–liquid extraction, etc.) showed great variations (from 3.14 ± 0.23 mg GAE/g dry sample to 637.3 ± 14.6 mg GAE/g dry sample), highlighting the fact that the phenolic content of roses, and in general, plant substrates, is affected by various parameters besides the extraction technique [12,13,14,15]. Taking into consideration these studies, the results of the extraction method optimized in the present work are satisfactory.

### 2.2. Total Phenolic Content, Antiradical and Antioxidant Activity of Rose Byproducts Extracts

The optimal UAE conditions were then applied in rose solid byproducts to investigate the perspectives of an upcoming re-valorization of the post-distillation residuals. Different rose byproducts were compared in terms of their total phenolic content and antioxidant and antiradical activity. Specifically, the Folin–Ciocâlteu, ABTS^•+^ and FRAP assays were performed in (a) post-distillation solid rose byproducts of different harvesting and distillation periods at optimal UAE conditions and at low phenolics UAE conditions (Run 3, Table S1) and (b) rose hydrosols (liquid byproducts). The results of the spectrophotometric methods are presented in Table 1.

Comparing the TPCs of rose raw samples and rose byproducts at the optimal UAE conditions, rose petals contained more phenolic compounds than rose solid residuals (Table 1). Rose byproducts are the remaining solid biomass after hydrodistillation, which is basically a thermal process of increased temperatures (about 100 °C). Therefore, the implementation of non-mild distillation conditions exhibit detrimental effects on the content of phenolic compounds [16].

As shown in Table 1, the UAE conditions that provided low TPC values using rose petals as substrate (Run 3, Table S1) also delivered extracts with low phenolic content when applied in rose solid byproducts, attesting to a further validation regarding the reliability and accuracy of the constructed Box–Behnken model. In addition, the different distillation period and therefore the different crop batches seem to affect the TPC of the optimal byproduct extracts (Table 1). Although the differences in the values of extracts’ antiradical and antioxidant activity were not so prominent compared to their TPC values, they follow the same trend and thus the extracts with high TPC also exhibited superior ABTS^•+^ and FRAP values. Nonetheless, according to the results of Pearson test, the correlation was moderate in the case of Folin-FRAP and Folin-ABTS^•+^ (r = 0.421 and r = 0.518, respectively) but also in the case of ABTS^•+^-FRAP (r = 0.567) insinuating the presence of compounds other than polyphenols that may act as antiradical and/or antioxidant agents. This result can be justified through the increased temperatures during hydrodistillation, which may promote the production of other compounds (i.e., Maillard reaction products) bearing antiradical and antioxidant properties [17] in the rose solid byproducts.

Even though TPC, ABTS^•+^, and FRAP values of hydrolates are not directly comparable to those of solid residuals due to distinct measurement units, these liquid byproducts could be further re-used for their antioxidant/antiradical properties, which in this case, are strongly related to the phenolic content of the hydrosols, as shown by the Pearson test (r Folin-FRAP = 0.858, r Folin-ABTS^•+^ = 0.827, r ABTS^•+^-FRAP = 0.995).

### 2.3. Interpretation of ATR-FTIR Spectrum of Rose Byproduct Extracts at Optimal UAE Conditions

The ATR-FTIR spectrum of rose byproducts at the best UAE conditions was recorded and interpreted in order to provide valuable insights regarding the phytochemical profile of rose solid byproducts targeting on their future valorization. The characterization and intensities of the spectral absorbance bands are exhibited in Table 2.

The interpretation of ATR-FTIR spectra confirmed the presence of phenolic and aromatic compounds, alcohols, amides or phenolic amide derivatives, sugars, and carbonyl compounds, in accordance with other studies focusing on FTIR flowers analyses [18,19,20,21,22].

### 2.4. Phenolic Fingerprint of Rose Byproducts Using Liquid Chromatography-Tandem Mass Spectrometry (LC-MS/MS) Information Dependent Acquisition (IDA) in Negative Ionization Mode

The LC-MS/MS analysis of the investigated solid byproduct extracts disclosed the presence of 13 phenolic compounds from the in-house library. The elucidated compounds are presented in Table 3. Indicative chromatographs of the phenolic compounds are illustrated in Figure S1.

According to Table 3, six phenolic acids, five flavonoids, one diphenol (pyrocatechol), and one hydrobenzaldehyde (syringaldehyde) were detected in rose solid byproducts. A literature lookup showed that our results were in line with the findings of other research groups that studied the phytochemical profile of *Rosa* species [13,23,24,25,26]. Nonetheless, the normalized contents of these compounds, as estimated by their *m*/*z* intensities, in the four examined extracts (Table S3), which will be further commented in the Section 3, were not similar, showing dependence from the extraction conditions and the harvesting and distillation time as well as the different rose batches [27,28,29]. Regarding the latest, it should be pointed out that catechin and pyrocatechol were only detected in the second batch of rose byproducts (RSB2_BEST), while rosmarinic acid, protocatehuic acid, and benzoic acid were absent from RSB2_LOW TPC, possibly due to the different growth stages or harvesting period.

## 3. Discussion

In the present study, ethanol content in the extracting agent and extraction time emerged as the most influential factors of UAE, followed by the quadratic effects of solvent-to-material ratio and US power. Focusing on ethanol content, the increase in the percentage in the water up to 60–70% [30,31] promoted the extraction of less polar phenolic compounds, such as non-polar flavonoids, which are the main group of phenolic molecules in many rose species, especially the edible ones [32]. However, ethanol percentages over 80% thwarted the diffusion of phenolic compound in the solvent [30]. In general, high TPCs could be achieved also with lower ethanol contents (~30%) if high extraction temperature is applied (over 60 °C) [33]. In addition, according to Koczka et al. (2018) [34], ethanolic extracts of *Rosa* species exhibited higher antioxidant properties than water extracts, confirming that hydroalcoholic solvents with increased ethanol content manage to recover more antioxidant compounds.

Furthermore, the extraction time normally affects the phenolic compounds yield, while it is vice versa related to the extraction temperature. Short extraction times combined with higher temperatures increase the extraction yield, whilst prolonged extraction periods with high temperatures may lead to the degradation, hydrolysis or oxidation of phenolic components [35]. When the sonication time is increased up to a level, the cavitation phenomenon is enhanced, facilitating the rupture of plant cells and the release of target analytes in the extraction solvent. Nevertheless, extended ultrasonic treatments result in the decline of TPC values, due to the possible deterioration of phenolic compounds [36,37]. Although the optimal extraction time is markedly dependent on the type of substrate and the analytes of interest, recent literature reported that phenolic acids and flavonoid were effectively extracted at ultrasonic exposure times around 20–40 min when temperature was relatively low (lower than 50 °C) and that the obtained extracts showed great antioxidant activities [35,38]. These outcomes are in line with the results of our work, where UAE performance in terms of TPC was maximized at 25 min and ambient temperature.

Ultrasonic power is strongly associated with various parameters of the UAE process, such as extraction time, temperature, and extraction solvent. Basically, higher US power intensifies the cavitation bubbles collapse and therefore enables the destruction of plant tissues and the diffusion of phenolic compounds. Moreover, the mechanical effects caused by US application (i.e., mechanical vibrations of US probe) expand the contact area between the matrix and the extraction solvent, facilitating the penetration of the substrate cells by the solvent. However, the excessive formation of cavitation bubbles caused by increased US intensities (which could provoke the degradation of the extracted compounds) may impede the transfer of US energy to the sample and therefore reduce the extraction efficiency, due to the ‘overpopulation’ of cavitation bubbles in the extraction solution and their insufficient collapse [37]. Thus, medium US power, as the one implemented in the present study (53%) is considered ideal for the acquisition of extracts rich in the desired solutes.

On the other hand, intermediate values of solid-to-material ratio (SMR), such as 30–40 mL/g, deliver the optimal results regarding the mass transfer from the sample to the extraction medium, since low SMRs do not accelerate the US cavitation due to high viscosity of the solvent solution, while the extremely high SMRs overpromote the cavitation phenomenon and result in the degradation of the targeted molecules [37].

Particular attention should be devoted to the high antioxidant values of the rose byproducts’ samples of low TPC (Table 1). These extracts were produced (Table S1) by applying lower ethanol percentage in the solvent solution (60% instead of 71% at the optimal UAE conditions) and higher extraction times (40 min instead of 25 min at the optimal UAE conditions). According to other studies, the FRAP values were increased with (a) the decrease of ethanol content, possibly due to the co-extraction of more polar compounds, which may act as more potent antioxidant agents [39] and with (b) the relative increase of extraction time, which establish the equilibrium between solid matrix and extraction solvent [40]. However, the ultrasonication of the extracts for more than 50 min prompted the reduction of FRAP values [41].

**Figure 4:**
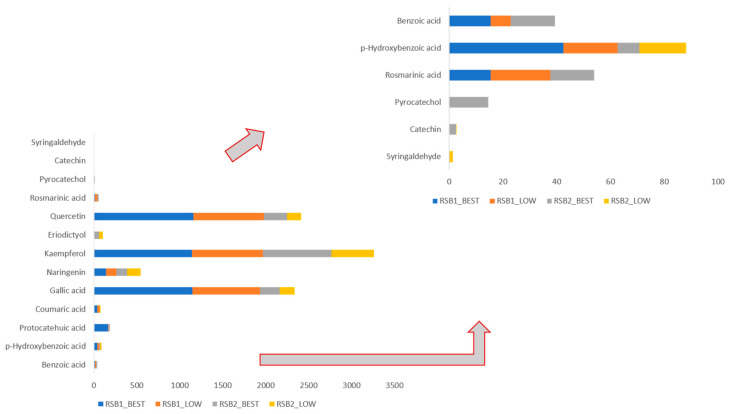
Normalized contents of identified phenolic compound by LC-MS/MS.

To take a step further, the variability in the extraction conditions of the investigated rose extracts, along with other parameters (i.e., time of harvesting, climatic conditions, etc.), also have an impact on shaping the phenolic fingerprint of the obtained extracts. The normalized contents of the identified phenolic constituents are depicted in Figure 4 and Table S3.

According to Figure 4 and Table S3, all extracts were rich in flavonoids (i.e., quercetin, naringenin, kaempferol), a fact that can be ascribed to the high percentage of ethanol in the solvent solution, which favors the recovery of compounds of less polar compounds of higher molecular weight. Furthermore, the most abundant phenolic acid in all extracts was gallic acid, followed by p-hydoxybenzoic acid. The solubility of these two compounds is increased when the content of alcohol in the solvent is also increased [42,43]. Other phenolic acids, such as protocatehuic, rosmarinic, and benzoic acids, were detected in lower amounts compared to flavonoids.

However, the assessment of LC-MS/MS spectra unveiled that harvesting period played a key role, even more significant than extraction conditions, in the phenolic fingerprint of rose extracts. For instance, the post-distillation extracts generated by roses harvested in early May (RSB1) contained higher amounts in most of the elucidated phenolic compounds (Figure 4) compared to the late May extracts (RSB2). Recent studies confirm that various pre-harvest and harvest parameters, such as different flowering and vegetative stages, harvesting at different time parts of the day (i.e., morning vs. afternoon), climatic and environmental conditions during blossom and harvesting (i.e., abiotic stress), genetic factors, impact on the qualitative and quantitative profile of the phenolic compounds. Even though the optimum harvesting period by means of TPC is tightly dependent on the plant, the harvesting of aromatic plants and flowers in earlier time (RSB1 vs. RSB2) usually results in higher TPCs, since in the beginning of flowering the metabolic mechanisms and pathways of the plant are dedicated on the production of volatile compounds, which will provide plant oil products with superior aroma composition [24,44,45].

Additionally, the interpretation of LC-MS/MS spectra explains the pattern of the FRAP and ABTS^•+^ values (Table 1), since certain in vitro activities of the extracts, such as the antiradical, antioxidant, and antimicrobial activities, are attributed not only in the quantity but also the type of the identified compounds. Thus, in spite of the fact that the rose extracts contained lower amounts of phenolic acids than flavonoids, the elucidated acids may register stronger antioxidant or antimicrobial potentials. Kędzierska-Matysek et al. (2021) attempted to correlate the antioxidant and antiradical activity of Polish honeys with specific phenolic compounds and reported that p-hydroxybenzoic acid and p-coumaric acid were strongly correlated to FRAP and ABTS^•+^, while quercetin and kaempferol present a very poor, even negative, correlation [46]. The structural information supplied by ATR-FTIR analysis (Table 2) attests the trend observed in spectrophotometric assays, since several aromatic, phenolic, and alcohol-related bands were reported. Moreover, the bands that correspond to amides presented the higher FTIR intensities (Table 2). Based on recent data [47,48,49], amides or phenolic amides derivatives were delineated as active radical scavengers with excellent antioxidant activities; therefore, amides compounds present in rose extracts could add to the FRAP and ABTS^•+^ values.

To conclude, solid post-distillation byproducts may serve as alternative functional ingredients of natural origin, which will be implemented or added in various food products, such as meat products, in the future.

## 4. Materials and Methods

### 4.1. Plant Material

Rose flowers of *Rosa Damascena*, commonly known as Damask rose, were cultivated in an open field in Voio, Kozani region (West Macedonia, Greece) and collected in May 2022, in two different harvesting periods (early May and late May). Two hundred kilos (200 kg) of rose petals were subjected to hydro-distillation for 4.30 h using 1000 L of water, to extract the essential oils of the plant. The byproducts of distillation process were then divided into the liquid fraction (hydrosol or aromatic waters) and the solid fraction (solid byproducts/residues or waste biomass). Post-distillation solid residues, which contained 93.6.% (on a wet basis) moisture and 0.995 water activity, were lyophilized (Thermo Scientific ModulyoD Freeze Dryer, Thermo Scientific, Waltham, MA, USA) until dryness and then stored at -20 °C until further analysis. All samples of rose petals and rose byproducts were provided by Kozani Roses (https://kozaniroses.gr/, accessed on 14 August 2023).

### 4.2. Ultrasound-Assisted Extraction (UAE) for the Recovery of Phenolic Compounds from Rose Samples and Byproducts

Ultrasound-assisted extraction (UAE) was implemented to the solid post-distillation byproducts for the recovery of phenolic compounds. The UAE process was performed by Sonoplus HD 4400 (Bandelin Sonoplus, Berlin, Germany) system with maximum ultrasonic nominal power of 400 W, equipped with an ultrasonic probe. For the extraction of phenolic compounds, different volumes of hydroethanolic solutions of various compositions were added to 0.5 g of rose dry biomass. Ethanol was preferred over methanol due to its equally high extraction yields of polyphenols, the lower toxicity and its biodegradability. The extraction vessels were immersed into an ice-cold bath throughout the extraction process to maintain the extraction temperature constant at 20–25 °C. The sonication of the samples was continuous in all cases. Next, the extracts were centrifuged at 3500 rpm for 15 min and the supernatant was kept for further analysis.

### 4.3. Implementation of Box–Behnken Design

The optimization of UAE was performed by applying a symmetrical 27-run three-level Box–Behnken design (BBD). The ethanol content, A (% *v*/*v*), the extraction time, B (minutes), the solvent-to-material ratio, C (mL/g), and the ultrasound (US) power (%), D were the factors under optimization or independent variables, while the total phenolic content (TPC), expressed as mg of gallic acid equivalents (GAE) per gram of dry sample, was the response of the model or dependent variable. The optimization process was conducted using as substrate the petals of the rose flower prior to distillation due to the higher amounts available for extraction, compared to those of their post-distillation residues.

Since the examined factors have different natural units, their actual values should be transformed in dimensionless normalized coded units (−1, 0, +1) to directly compare and evaluate their effect and the effect of their interactions on the TPC. The actual and coded values of the investigated extraction factors are presented in Table 4.

### 4.4. Spectrophotometric Assays

The total phenolic content (TPC) of the rose samples was determined using a modified version of the Folin–Ciocâlteu assay. The measurements were conducted in triplicate using a Spectro 23 Digital Spectrophotometer (Labomed, Inc., Los Angeles, CA, USA). The absorbance was measured at 750 nm, while the results were reported as milligrams of gallic acid equivalents (GAE) per 1 g of dried rose samples. Standard solutions with a concentration range of 20–500 mg/L gallic acid were utilized for the calibration curve [50].

To assess the antiradical activity of the rose samples against the ABTS^●+^ radical, the method described by Lantzouraki et al. (2015) [51] was employed. Measurements were taken at 734 nm, and the antiradical activity was expressed as milligrams of Trolox Equivalents (TE) per 1 g of dried rose samples. Standard solutions ranging from 0.20 to 1.5 mM of Trolox were used for the construction of the calibration curve.

The Ferric Reducing Antioxidant Power (FRAP) assay, based on the technique by Lantzouraki et al. (2015) [52], was performed to determine the antioxidant activity. Absorbance readings were taken at 595 nm. The antioxidant activity was reported as milligrams of Fe(II) equivalents per 1 g of dried rose samples. Standard solutions with a concentration range of 600–2000 μM of FeSO_4_·7H_2_O were employed for the calibration curve.

### 4.5. Attenuated Total Reflectance-Fourier Transform Infrared Spectroscopy (ATR-FTIR) Analysis

The FTIR spectrum was registered at room temperature by applying attenuated total reflectance (ATR). Τhe dried residues of rose samples were loaded in an FTIR spectrometer (Shimadzu, IRAffinity-1S FTIR Spectrometer, Kyoto, Japan). The reference for ATR was adjusted at 3284.77 cm^−1^, while the samples and the background spectra were acquired from 4000 to 499 cm^−1^. In addition, the average of 20 scans at a resolution of 4 cm^−1^ was documented. Data processing and analysis were performed using LabSolutions IR software (version 2.21, Shimadzu, IRAffinity-1S FTIR Spectrometer, Kyoto, Japan) [21].

### 4.6. Phenolic Profile of the Extracts by Using Liquid Chromatography-Tandem Mass Spectrometry (LC-MS/MS)

For the chromatographic separation and mass spectral identification of phenolic compounds a liquid chromatography-tandem mass spectrometry (LC-MS/MS) method, previously developed by our research group [53], was applied for the assessment of the phenolic profile of rose extracts. In particular, 1 mL of rose extract (rose petals or rose distillation byproducts) was lyophilized and the dried residues were re-dissolved in 1000 μL of LC-MS grade methanol +0.1% *v*/*v* formic acid. Prior to LC-MS/MS analysis, all samples were filtered using Chromafil Xtra PET 0.45 μm (Macherey-Nagel, Düren, Germany).

The chromatographic system consists of an Agilent Eclipse Plus C-18 reversed-phase column (50 mm × 2.1 mm inner diameter, 3.5 µm particle size) linked with a RRLC in-line filter kit (2.1 mm, 0.2 µm filter) (Agilent Technologies, Santa Clara, CA, USA), while water +0.2% *v*/*v* formic acid (Solvent 1) and acetonitrile +0.1% *v*/*v* formic acid (Solvent 2) were the mobile phase binary solvent system. The gradient elution program and the flow rate alterations are described in detail in the work of Kavga et al. (2018) [54]. The temperature of the autosampler and column was set at 25 °C and the injection volume was 5 μL.

For the mass spectral analysis, a 3200 Q TRAP triple-quadrupole linear ion trap mass spectrometer (Sciex, Framingham, MA, USA) with electrospray ionization (ESI) source in negative ionization mode. The elucidation of phenolics compounds in rose extracts was performed by information dependent acquisition (IDA)-triggered MS/MS scans (EPI—enhanced product ion scans) based on an in-house built library of the 40 phenolic standards, as described by Kavga et al. (2018) [54] and by Tsiaka et al. (2022) [55], at a mass tolerance of 5 ppm for the MS/MS analysis. All data were processed by the Analyst software (version 1.6.2) (Sciex, Framingham, MA, USA).

### 4.7. Statistical Analysis

The data processing and the statistical analysis was performed using Minitab software (trial version, Minitab LCC., State College, PA, USA). All measurements were carried out at a confidence level of 95% (*p*-value ≤ 0.05).

## 5. Conclusions

As the plant EOs industry is relentlessly flourishing, a huge amount of post-distillation byproducts, mostly solid biomass, is piling up, without meeting any obvious or direct use. Besides the ecological issues, the accumulation of plant biomass, rich in bioactive compounds, consists of a foremost economic burden, due to the high-cost management strategies that should be implemented. In this context, the valorization of plant byproducts through their conversion into valuable co-products and therefore, their re-use is of utmost importance.

Although complementary studies should be performed to delineate the use of rose byproducts extracts, the results of the present study can set different routes, since it provides further information regarding the effect of UAE extraction conditions on the antioxidant/antiradical activity and on the phenolic fingerprint of the extracts, shifting the focus towards the production of fitted-to-application rose extracts.

## Figures and Tables

**Table 1:** Total phenolic content, antiradical and antioxidant activity of various types of rose samples.

Sample	Sample Code Name	Average TPC (mg GAE/g Dry Sample) (±stdev) ^1^, N = 3 ^2^	Average ABTS^•+^ (mg of TE/g of Dry Sample) (±stdev) ^1^, N = 3 ^2^	Average FRAP (mg of Fe (II)/g of Dry Sample) (±stdev) ^1^, N = 3 ^2^
Rose solid byproducts_15/05/2022 *_ Optimal UAE conditions	RSB1_BEST	56.7 (±0.36) ^a^	414 (±57) ^b^	604.5 (±2.8) ^a^
Rose solid byproducts_15/05/2022_ Low phenolics UAE conditions	RSB1_LOW TPC	5.22 (±0.36) ^c^	113 (±40) ^c^	414 (±44) ^b^
Rose solid byproducts_30/05/2022_ Optimal UAE conditions	RSB2_BEST	23.6 (±0.27) ^b^	842 (±48) ^a^	601 (±31) ^a^
Rose solid byproducts_30/05/2022_ Low phenolics UAE conditions	RSB2_LOW TPC	3.13 (±0.28) ^c^	164.0 (±9.1) ^c^	554 (±117) ^a^
		**Average TPC (mg GAE/L of liquid sample) (±stdev) ^1^, N = 3 ^2^**	**Average ABTS** ^•+^ **(mg of TE/L of liquid sample) (±stdev) ^1^, N = 3 ^2^**	**Average FRAP (mg of Fe (II)/L of liquid sample) (±stdev) ^1^, N = 3 ^2^**
Hydrosol_30/05/2022	RLB1	1038 (±13) ^A^	3865 (±195) ^A^	8838 (±106) ^A^
Hydrosol_16/06/2022	RLB2	726 (±70) ^B^	1268 (±75) ^B^	3471 (±227) ^B^

* date of distillation; ^1^ stdev: standard deviation; ^2^ number of replicates; ^a–c^ and ^A,B^: Different letters in the same column (method) denote significant difference (*p*-value ≤ 0.05) between the samples.

**Table 2:** Characteristic spectral absorbance bands of optimal rose byproduct extracts.

Regions (cm^−1^)	Band Annotation	Intensities
745–705	Bending vibration of C-H at the CH_2_ of m-disubstituted aromatic derivatives (aliphatic rocking vibrations)	0.019
810–750	Stretching vibration C_aromatic_-H in m-disubstituted aromatic derivatives	0.030
860–800	Stretching vibration C_aromatic_-H in o-disubstituted aromatic derivatives	0.055
900–860	Stretching vibration C_aromatic_-H in m-disubstituted aromatic derivatives	0.124
1020–1045	Stretching vibration C-O of glycoside bond in sugars	0.240
1050–1080	Stretching vibration of C-O ether bond in primary alcohols	0.032
1100–1120	Stretching vibration of C-O ether bond in secondary alcohols	0.026
1150–1170	Stretching vibration of C-O ether bond in tertiary alcohols or proteins	0.029
1170–1200	Stretching vibration of C-O ether bond in phenolic compounds	0.123
1270–1230	Stretching vibration of C-O ether bond	0.035
1380–1360	Bending vibration O-H of the C-OH group	0.020
1410–1310	Bending vibration O-H in phenols or tertiary alcohols	0.024
1470–1430	Bending vibration of C-H bond in methyl or methylene	0.033
1530–1500	Aromatic band	0.016
1650–1550	Bending vibration of >N-H secondary amino groups	0.031
1700–1600	Stretching vibration of carbonyl C=O in amides	0.297
1725–1720	Stretching vibration of carbonyl C=O in carboxylic acids and carbonyl compounds	0.029
2855	Symmetric stretching vibration C-H bond in CH_3_ methyl and CH_2_ methylene groups	0.011
2922	Asymmetric stretching vibration C-H bond in CH_3_ methyl and CH_2_ methylene groups	0.373
3500–3200	Stretching vibration of alcohols OH	0.031
3640–3530	Stretching vibration of phenolic compounds OH	0.030

**Table 3:** Identified phenolic compounds in rose solid byproducts by the LC-MS/MS analysis.

Phenolic Compound	RSB1_BEST	RSB1_LOW TPC	RSB2_BEST	RSB2_LOW TPC
Benzoic acid	√	√	√	
Catechin			√	√
Coumaric acid	√	√	√	√
Eriodictyol			√	√
Gallic acid	√	√	√	√
Kaempferol	√	√	√	√
Naringenin	√	√	√	√
Pyrocatechol			√	√
Protocatehuic acid	√	√	√	
Quercetin	√	√	√	√
Rosmarinic acid	√	√	√	
Syringaldehyde				√
p-Hydroxybenzoic acid	√	√	√	√

√: the symbol states the presence of a compound in each sample.

**Table 4:** Actual and coded values of the investigated factors.

Extraction Factors	Coded Values/Real Values		
	−1	0	+1
Ethanol content (A, % *v*/*v*)	60	80	100
Extraction time (B, min)	10	25	40
Solvent-to-material ratio (C, mL/g)	20	40	60
US power (D, %)	20	50	80

## Data Availability

Not applicable.

## Supplementary Materials

The following supporting information can be downloaded at: https://www.mdpi.com/article/10.3390/molecules28217403/s1, Figure S1: Chromatographs of selected identified phenolic compounds; Table S1: Total phenolic content of the experimental runs proposed by the Box–Behnken design; Table S2: ANOVA table of the applied Box–Behnken design. Table S3: Intensities of the phenolic compounds elucidated by LC-MS/MS analysis.
