# Supplementary material for: Optimization of Ultrasound-Assisted Extraction and Characterization of the Phenolic Compounds in Rose Distillation Side Streams Using Spectrophotometric Assays and High-Throughput Analytical Techniques
Source: Molecules. 2023 Nov 2;28(21):7403. doi: 10.3390/molecules28217403 (PMC10650874; doi:10.3390/molecules28217403)
Supplement: Supplementary file 1 [file molecules-28-07403-s001.zip › molecules-2647396-supplementary.pdf]

Table S1. Total phenolic content of the experimental runs proposed by the Box-Behnken design.

Experimental run	Ethanol content (% v/v)	Extraction time (minutes)	Solvent/material ratio (mL/g)	US power (%)	TPC (mg GAE/g dry sample) (±stdev)*, N=3[1]
21	80	10	40	20	73.6(±3.1)
18	100	25	20	50	9.1(±1.6)
4	100	40	40	50	24.0(±1.2)
25	80	25	40	50	141.1(±3.8)
11	60	25	40	80	140(±18)
6	80	25	60	20	84.4(±1.4)
13	80	10	20	50	90.3(±1.8)
14	80	40	20	50	104.3(±8.9)
9	60	25	40	20	126.3(±2.0)
22	80	40	40	20	143(±12)
1	60	10	40	50	81.5(±7.0)
26	80	25	40	50	127(±11)
20	100	25	60	50	13.8(±4.6)
16	80	40	60	50	143(±22)
17	60	25	20	50	145(±16)
24	80	40	40	80	129(±13)
7	80	25	20	80	114(±17)
8	80	25	60	80	89(±17)
10	100	25	40	20	2.8(±1.4)
23	80	10	40	80	111(±11)
27	80	25	40	50	141(±12)
12	100	25	40	80	20.05(±0.78)
15	80	10	60	50	77(±10)
2	100	10	40	50	22.5(±1.8)
19	60	25	60	50	126(±14)
5	80	25	20	20	101.1(±7.5)
3	60	40	40	50	145(±7.2)

*stdev: standard deviation; [1] number of replicates

Table S2. ANOVA table of the applied Box-Behnken design.

Source	DF	Adj SS	Adj MS	*F*-Value	*p*-Value
Model	11	58432.6	5312.1	36.45	0.000
Linear terms	4	42868.8	10717.2	73.53	0.000
Ethanol content (%) (A)	1	37746,5	37746.5	258.99	0.000
Extraction time (min) (B)	1	4610.0	4610.0	31.63	0.000
Solvent/material (mL/g) (C)	1	80.9	80.9	0.56	0.468
US power (%) (D)	1	431.3	431.3	2.96	0.106
Square terms	4	13201.0	3300.2	22.64	0.000
Ethanol content (%)*Ethanol content (%) (AA)	1	13041.4	13041.4	89.48	0.000
Extraction time (min)*Extraction time (min) (BB)	1	961.7	961.7	6.60	0.021
Solvent/material (mL/g)*Solvent/material (mL/g) (CC)	1	1982.1	1982.1	13.60	0.002
US power (%)*US power (%) (DD)	1	1135.5	1135.5	7.79	0.014
2-Way Interaction	3	2362.9	787.6	5.40	0.010
Ethanol content (%)*Extraction time (min) (AB)	1	974.4	974.4	6.69	0.021
Extraction time (min)*Solvent/material (mL/g) (BC)	1	709.8	709.8	4.87	0.043
Extraction time (min)*US power (%) (BD)	1	678.6	678.6	4.66	0.048
Error	15	2186.2	145.7		
Lack-of-Fit	13	2069.8	159.2	2.74	0.299
Pure Error	2	116.4	58.2		
Total	26	60618.8			
R^2	0.964				
R^2_{adj}	0.938				
R^2_{pred}	0.854				

Table S3. Intensities of the phenolic compounds elucidated by LC-MS/MS analysis.

	m/z intensities of the elucidated compounds ($cps \times 10^6$)			
**Phenolic compound**	**RSB1_BEST**	**RSB1_LOW TPC**	**RSB2_BEST**	**RSB2_LOW TPC**
Benzoic acid	15.47	7.45	16.47	-
Catechin	-	-	2.58	1.30
Coumaric acid	38.68	30.37	-	7.45
Eriodictyol	-	-	62.14	40.60
Gallic acid	1148.94	786.98	226.80	173.96
Kaempferol	1141.20	828.40	795.34	497.04
Naringenin	145.84	117.91	124.27	156.57
Pyrocatechol	-	-	14.60	-
Protocatehuic acid	162.46	6.35	18.64	-
Quercetin	1160.54	817.36	274.33	160.16
Rosmarinic acid	15.47	22.09	16.47	-
Syringaldehyde	-	-	-	1.30
p-Hydroxybenzoic acid	42.55	20.16	8.06	17.40

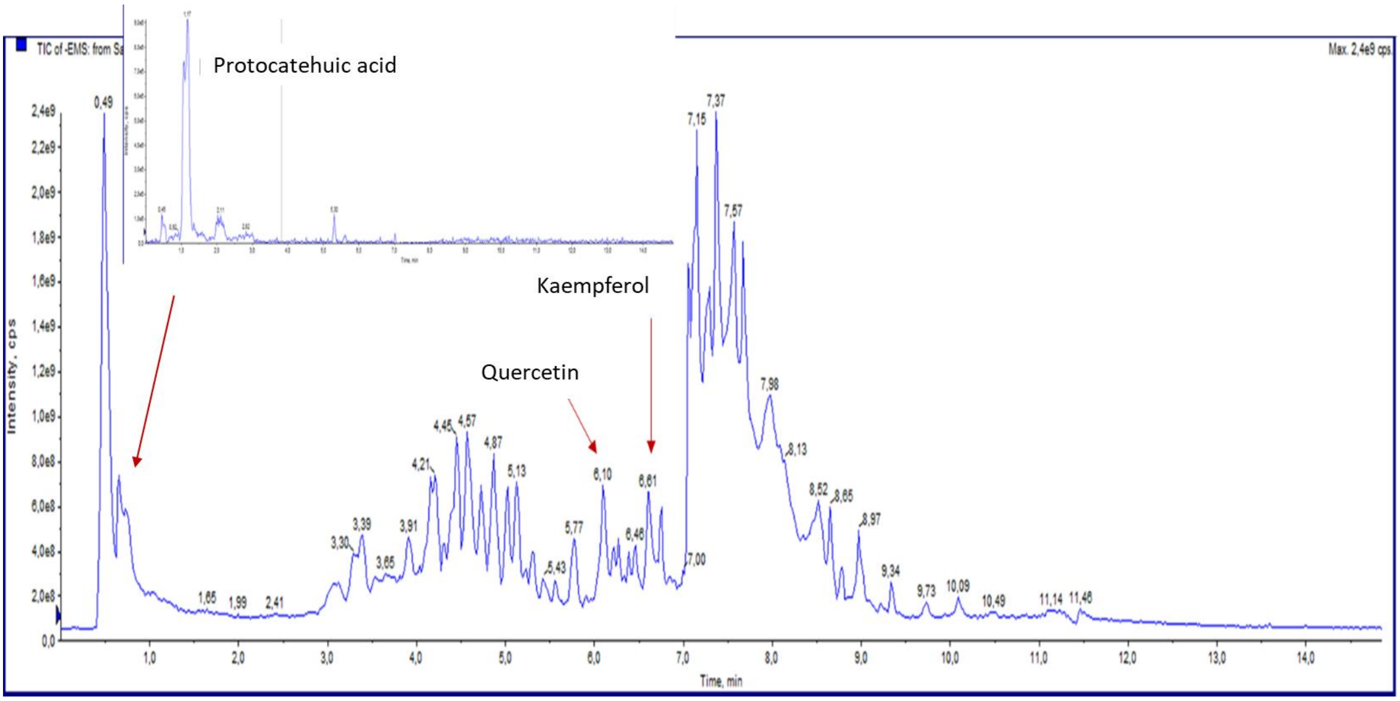

Figure S1. Chromatographs mass spectra of selected identified phenolic compounds.
